# Bi-directional astrocytic regulation of neuronal activity within a network

**DOI:** 10.3389/fncom.2012.00092

**Published:** 2012-11-02

**Authors:** S. Yu Gordleeva, S. V. Stasenko, A. V. Semyanov, A. E. Dityatev, V. B. Kazantsev

**Affiliations:** ^1^Laboratory of Nonlinear Dynamics of Living Systems, Institute of Applied Physics of Russian Academy of ScienceNizhny Novgorod, Russia; ^2^Laboratory of Brain Extracellular Matrix Research, University of Nizhny NovgorodNizhny Novgorod, Russia; ^3^RIKEN Brain Science InstituteSaitama, Japan; ^4^Department of Neuroscience and Brain Technologies, Istituto Italiano di TecnologiaGenova, Italy

**Keywords:** neuron, astrocyte, synaptic transmission, tripartite synapse, neuronal network, regulation

## Abstract

The concept of a tripartite synapse holds that astrocytes can affect both the pre- and post-synaptic compartments through the Ca^2+^-dependent release of gliotransmitters. Because astrocytic Ca^2+^ transients usually last for a few seconds, we assumed that astrocytic regulation of synaptic transmission may also occur on the scale of seconds. Here, we considered the basic physiological functions of tripartite synapses and investigated astrocytic regulation at the level of neural network activity. The firing dynamics of individual neurons in a spontaneous firing network was described by the Hodgkin–Huxley model. The neurons received excitatory synaptic input driven by the Poisson spike train with variable frequency. The mean field concentration of the released neurotransmitter was used to describe the presynaptic dynamics. The amplitudes of the excitatory postsynaptic currents (PSCs) obeyed the gamma distribution law. In our model, astrocytes depressed the presynaptic release and enhanced the PSCs. As a result, low frequency synaptic input was suppressed while high frequency input was amplified. The analysis of the neuron spiking frequency as an indicator of network activity revealed that tripartite synaptic transmission dramatically changed the local network operation compared to bipartite synapses. Specifically, the astrocytes supported homeostatic regulation of the network activity by increasing or decreasing firing of the neurons. Thus, the astrocyte activation may modulate a transition of neural network into bistable regime of activity with two stable firing levels and spontaneous transitions between them.

## Introduction

Determining the principles of signal processing in brain networks has been one key challenge in modern neuroscience, which has thus far been unresolved. A central mechanism of signal propagation is synaptic transmission between neurons constituting networks. There is evidence that in addition to processes within the pre- and post-synaptic compartments, several extrasynaptic signaling pathways can affect this transmission (Semyanov, 2008; Dityatev and Rusakov, 2011), one of which is the influence of neighboring astrocytes modulating synaptic signaling. The idea of astrocytes being important in addition to the pre- and post-synaptic components of the synapse has led to the concept of a tripartite synapse (Araque et al., 1999; Haydon, 2001). A part of the neurotransmitter released from the presynaptic terminals (i.e., glutamate) can diffuse out of the synaptic cleft and bind to metabotropic glutamate receptors (mGluRs) on the astrocytic processes that are located near the neuronal synaptic compartments. The neurotransmitter activates G-protein mediated signaling cascades that result in phospholipase C (PLC) activation and insitol-1,4,5-trisphosphaste (IP3) production. The IP3 binds to IP3-receptors in the intracellular stores and triggers Ca^2+^ release into the cytoplasm. Such an increase in intracellular Ca^2+^ can trigger the release of gliotransmitters (Parpura and Zorec, 2010) [e.g., glutamate, adenosine triphosphate (ATP), D-serine, and GABA] into the extracellular space.

A gliotransmitter can affect both the pre- and post-synaptic parts of the neuron. By binding to presynaptic receptors it can either potentiate or depress presynaptic release probability. One of the key pathways in tripartite synapse is mediated by glutamate released by the astrocyte (Parri et al., 2001; Liu et al., 2004a,b; Perea and Araque, 2007). Such glutamate can potentially target presynaptic NMDA receptors which increase release probability (McGuinness et al., 2010), or presynaptic mGluRs which decrease it (Semyanov and Kullmann, 2000). Presynaptic kainate receptors exhibit a more complex modulation of synaptic transmission through both metabotropic and ionotropic effects (Semyanov and Kullmann, 2001; Contractor et al., 2011).

In addition to presynaptic feedback signaling through the activation of astrocytes, there is feedforward signaling that targets the postsynaptic neuron. Astrocytic glutamate induces slow inward postsynaptic currents (SICs) (Parpura and Haydon, 2000; Parri et al., 2001; Fellin et al., 2006). Their appearance is characterized by a high-degree of spatial and temporal correlation in different cells, thus producing a synchronization effect (Fellin et al., 2006). Astrocytic release of D-serine is critical for the activation of postsynaptic NMDA receptors and the development of synaptic long-term potentiation (LTP) (Henneberger et al., 2010; Bergersen et al., 2011). In contrast, GABA released by astrocytes may be responsible for synchronous inhibition of postsynaptic neurons (Liu et al., 2000; Kozlov et al., 2006; Angulo et al., 2008). Another gliotransmitter, ATP, can also directly depress the postsynaptic neuron by activating purinergic receptors (Koizumi et al., 2003). Additionally, ATP can increase the spike generation probability in interneurons through activation of the P2Y1 receptors (Fellin et al., 2006; Torres et al., 2012). Thus, astrocytes may play a significant role in regulation of neuronal network signaling by forming local elevations of gliotransmitters that can guide excitation flow (Semyanov, 2008; Giaume et al., 2010). By integration of neuronal synaptic signals, astrocytes provide coordinated release of gliotransmitters affecting local groups of synapses from different neurons. This action may control the level of coherence in synaptic transmission in neuronal groups (for example, by means of above mentioned SICs). Moreover, different astrocytes are coupled by gap junctions and may be able propagate such effect even further by means intercellular IP3 and Ca^2+^ diffusion (Verkhratsky and Butt, 2007). Moreover the astrocytes can communicate to each other by extracellular ATP diffusion. Thus, theoretically the astrocytes may contribute in regulation of neuronal activity between distant network sites.

Several mathematical models have been proposed to understand the functional role of astrocytes in neuronal dynamics: a model of the “dressed neuron,” which describes the astrocyte-mediated changes in neural excitability (Nadkarni and Jung, 2004, 2007), a model of the astrocyte serving as a frequency selective “gate keeper” (Volman et al., 2006), and a model of the astrocyte regulating presynaptic functions (De Pittà et al., 2011). It has been demonstrated that gliotransmitters can effectively control presynaptic facilitation and depression. The model of the tripartite synapse has recently been employed to demonstrate the functions of astrocytes in the coordination of neuronal network signaling, in particular, spike-timing-dependent plasticity and learning (Postnov et al., 2007; Amiri et al., 2011; Wade et al., 2011). In models of astrocytic networks, communication between astrocytes has been described as Ca^2+^ wave propagation and synchronization of Ca^2+^ waves (Ullah et al., 2006; Kazantsev, 2009). However, due to a variety of potential actions, that may be specific for brain regions and neuronal sub-types, the functional roles of astrocytes in network dynamics are still a subject of debate.

In this paper we illustrate how activations of local astrocytes may effectively control a network through combination of different actions of gliotransmitters (presynaptic depression and postsynaptic enhancement). We found bi-directional frequency dependent modulation of spike transmission frequency in a network neuron. A network function of the neuron implied the presence of correlation between neuron input and output reflecting feedback formed by synaptic transmission pathways. Surprisingly, the bi-directional astrocytic regulation, which may be negligibly small for local synaptic transmission, may induce significant changes in network firing states, including the appearance of rate-encoded bistable states.

## Materials and methods

To study astrocytic regulation of neuronal activity, we introduced a computational model of synapses involved in spontaneous firing dynamics of a neuronal network using the mean field approach. We assumed that a spiking neuron is a member of a network, and the spikes of this neuron go through divergent/convergent connections of the network providing a certain level of correlation between neuron output and input. Because of complex network connectivity, it was impractical to follow the propagation of individual spikes, and thus we followed the evolution of the firing rates when the frequency was averaged in the time window of hundreds of milliseconds or seconds. We considered a postsynaptic neuron capable of spike generation when integrating the incoming postsynaptic currents (PSCs). These currents were treated as a mean field contribution of a large number of tripartite synapses. The presynaptic dynamics consisted of spontaneous glutamate release and the glutamate release induced by the network feedback. The presynaptic terminals were excited through different signaling pathways and thus were uncorrelated at the millisecond time scale. The astrocytic compartment represented a set of local processes that can independently modulate the transmission at particular synapses. Independent modulation of different synapses by the astrocyte is based on experimental observations that local Ca^2+^ sparks in astrocytic processes are independent from each other and different from the global Ca^2+^ transient that spread through the entire astrocyte (Nett et al., 2002). Thus, the feedback and feedforward actions of the particular astrocyte process were localized and related to a particular synapse in our model. The duration of Ca^2+^ sparks was a few seconds long, and each of them was associated with the local release of gliotransmitter. In the model, we assumed that these releases modulated synaptic transmission, but did not produced synaptic synchronization, which require whole astrocyte activation.

### Presynaptic dynamics

Each presynaptic event caused the release of a quantum of glutamate. Because the dynamics of the neurotransmitter was averaged from all the synaptic terminals at a relatively long time scale (up to seconds) we did not describe detailed presynaptic kinetics that operates in a short-time scale. The mean field amount of neurotransmitter, *X*, that diffused from synaptic cleft and reached the astrocyte was described by the following first-order equation,

(1) $$\begin{array}{l} \;\frac{dX}{dt}=-\alpha_{x}(X-k_{\text{pre}}H_{x}(I_{\text{pre}}-0.5)),\\ I_{\text{pre}}(t)=\{\begin{array}{cc} \begin{array}{l} 1,\\ 0, \end{array} & \begin{array}{l} \text{if}\;t_{i}<t<t_{i}+\tau ,\\ \text{otherwise}, \end{array} \end{array} \end{array}$$

where *I*_pre_(*t*) is a pulse signal accounting for the release events, *H*_*x*_ is the Heviside step function, *t*_*i*_ is the event occurrence time at one of the presynaptic terminals satisfying Poisson distribution with average Poisson frequency *f*_in_ and τ is the pulse duration, τ = 1 ms. Each presynaptic release event contributed to the concentration with the portion, Δ*X*_*i*_ ≈ (*k*_pre_ − *X*)α_*x*_, where *k*_pre_ is the efficacy of the release, and α_*x*_ is the neurotransmitter clearance constant. Thus, there was a temporal summation of the neurotransmitter amount released with the time scale α_*x*_. High frequency trains led to an increase in the mean field concentration. The amount of release varied with *X* to reflect the frequency-dependence of the release probability as demonstrated by Tsodyks and Markram in the short-term plasticity model (Tsodyks et al., 1998; De Pittà et al., 2011). We, however, did not include short-term plasticity in the model because the consequent pulses may indicate the release in spatially distinct synapses, which are averaged in Equation (1).

For sake of simplicity, our mean field model considers a set of independent (uncorrelated) events localized in different spatial sites as single averaged event. The diffusion processes are also accounted by effective average parameters α_*x*_ and *k*_pre_.

### Postsynaptic dynamics

The release of neurotransmitter leads to a PSC. We assumed that a number of events occurred in different spatial sites of dendritic tree were integrated at soma and provided mean field synaptic input, *I*_syn_, depolarizing the membrane that may lead to the response spike generation.

We focused on excitatory transmission and investigated excitatory postsynaptic currents (EPSCs), *I*_EPSCs_, using the following equation:

(2) $$\frac{dI_{\text{EPSCs}}}{dt}=\alpha_{I}(I_{\text{EPSCs}}-AH_{x}(I_{\text{pre}}-0.5)),$$

where α_*i*_ is their rate constant and *A* is their amplitude. Following experimental observations, we assumed that the amplitude of the EPSCs satisfy the probability distribution, *P*(*A*), in the following form:

(3) $$P(A)=\frac{2A}{b^{2}}\exp\left(-A^{2}/b^{2}\right),\;\int_{0}^{+\infty }P(A)dA=\Gamma (1)=1,$$

where Γ is the gamma function and *b* is the scaling factor that accounts for the effective strength of the synaptic input. Importantly, the synaptic events do not fully correlate with the consequent input pulses (action potentials) in the model because they can occur at different synaptic sites.

The postsynaptic events occurring at different sites of the dendritic tree are integrated and form synaptic current, *I*_syn_. Because we consider *I*_EPSCs_ (*t*) as a mean field contribution of all synapses, integrated synaptic current in the soma, *I*_syn_, can be expressed as:

(4) $$I_{\text{syn}}=I_{\text{EPSCs}}S(X),$$

where *S*(*X*) is a dendrite integration function expressed in the form of a high-pass filter that reflects the fact that the postsynaptic spike generation requires a summation of several synaptic inputs, e.g., single synaptic events will be filtered.

(5) $$S(X)=\frac{1}{1+\exp\left(-\frac{\left(X-\theta_{x}\right)}{k_{x}}\right)}$$

where θ_*x*_ and *k*_*x*_ are the midpoint and the slope of the neuronal activation, respectively.

**Figure 1:**
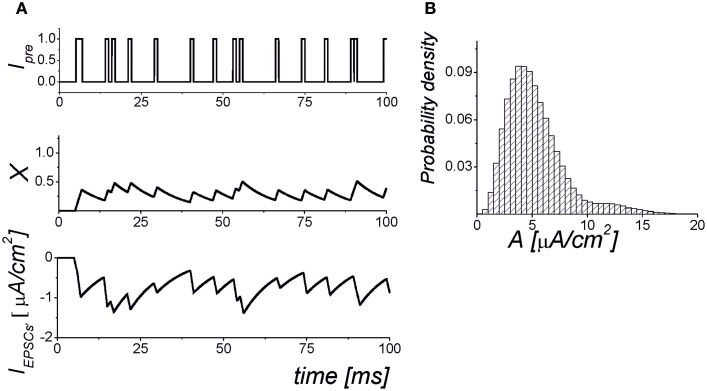
**The dynamics of the synaptic transmission model (1–6). (A)** Signaling *I*_pre_(*t*) models of presynaptic events in the form of the Poisson pulse train. Each pulse has a fixed duration, τ = 1 ms. The pulses exhibiting less than 1 ms intervals are considered as synchronized pulse events with a longer duration. The *X*(*t*) is the mean field concentration of neurotransmitter released for each pulse. The *I*_EPSC_ is mean field postsynaptic current with amplitudes selected according to the probability distribution shown in panel **(B)**. The parameter values: α_*x*_ = 0.05 ms^−1^, *k*_pre_ = 1, *b* = 25, θ_*x*_ = 0.35.

We modeled the spike generation with classical Hodgkin–Huxley equations (Hodgkin and Huxley, 1952). The membrane potential evolved according to the following current balance equation:

(6) $$C\frac{dV}{dt}=-(I_{\text{mem}}+I_{\text{th}}+I_{\text{syn}}),$$

where *I*_mem_ = *I*_Na_ + *I*_K_ + *I*_leak_ is the sum of the transmembrane currents responsible for the spike generation (for more details, see Izhikevich, 2007). Figure 1 illustrates the dynamics of synaptic transmission in Equations (1–6) obtained in numerical simulations.

**Figure 2:**
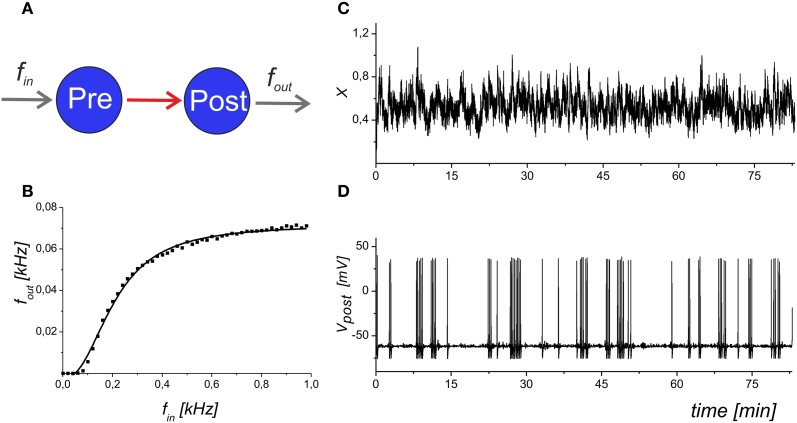
**The input–output dynamics of a neuron with synapses, but without astrocytic influence. (A)** Schematic illustration of synaptically coupled neurons with input frequency *f*_in_ and output spiking rate *f*_out_. **(B)** The dependence of the average firing rate, *f*_out_, averaged for 1 s, on the presynaptic event frequency. The solid line shows the logistic curve fit of the model data. **(C)** The mean field concentration of the neurotransmitter, *X*(*t*). **(D)** The output spike train that corresponds to the maximal slope of the frequency dependence. Parameter values: *f*_in_ = 0.2 kHz, *b* = 5.

We analysed the frequency of the spike generation, *f*_out_, depending on the input Poisson frequency, *f*_in_. Figure 2 illustrates the input–output characteristics of the spike transmission in Equations (1–6).

### Astrocytic dynamics

We added an astrocytic component to Equations (1–4) and assumed that gliotransmitters are released and act on the synapses. In the mean field model, we described the concentration of gliotransmitter by the following equation:

(7) $$\frac{dY_{k}}{dt}=-\alpha_{k}(Y_{k}-H_{k}(X)),\;H_{k}(X)=\frac{1}{1+\exp\left(-\frac{\left(X-\theta_{k}\right)}{k_{k}}\right)}.$$

**Figure 3:**
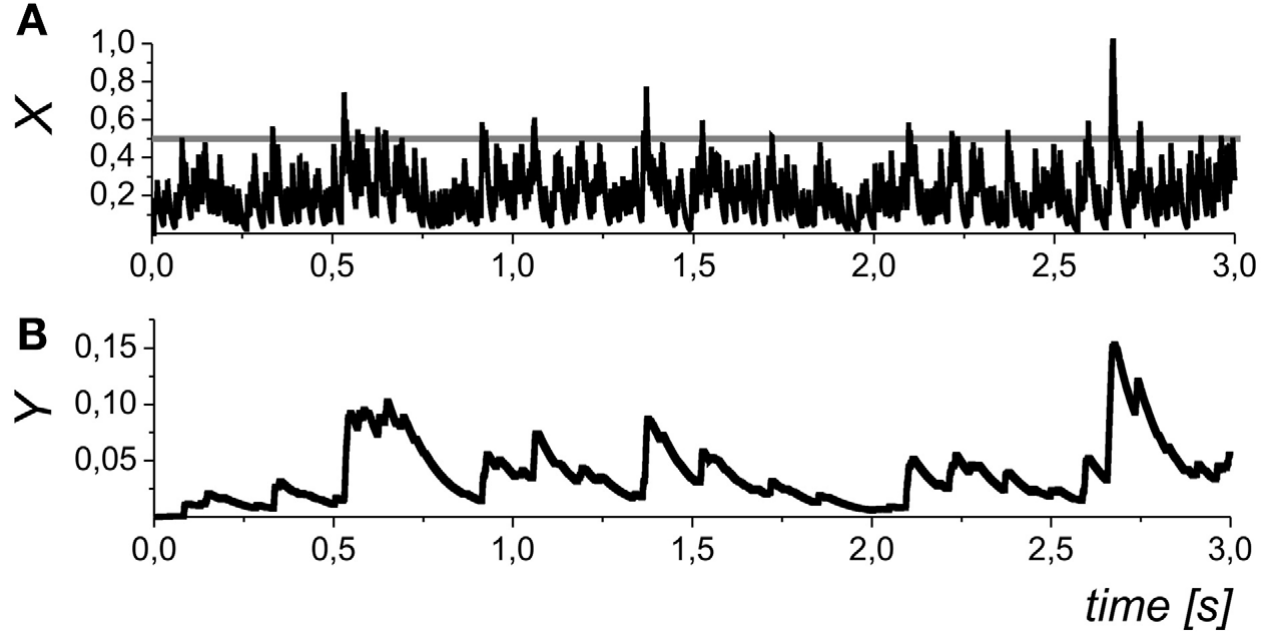
**The dynamics of the mean field concentration of neurotransmitter (A) and gliatransmitters (B) in Equations (5) and (10)**. The slow transients in the *Y*_*k*_ variables may be induced by different astrocytes (and/or different compartments of the same astrocytes), and, in general, they may have variable amplitudes. The gray line in the in panel **(A)** shows the midpoint of astrocyte activation function *H*_*k*_(*X*). Parameter values: θ_*k*_ = 0.5, and *k*_*k*_ = 0.01.

We assumed that different types of gliotransmitter (*k* = 1 for glutamate and *k* = 2 for D-serine released from astrocyte) may have different clearance rate, α_*k*_, and equilibrium activation function, *H*_*k*_(*X*), which accounts for the gliotransmitter amount released if the presynaptic activity exceeds a certain threshold (Perea and Araque, 2002) described here by the parameter θ_*k*_. Note, that Equation (7) is functionally similar to the gliotransmitter model that was recently proposed in (De Pittà et al., 2011) by excluding the computation of intracellular Ca^2+^ dynamics and focusing on neurotransmission modulation. The dynamics of the gliotransmitter concentration is illustrated in Figure 3.

In the mean field approach we did not set a definite limit for the duration of the astrocyte action. In addition to the neurotransmitter concentration, *X*, which accounts for the mean field impact of a number of synapses was variable, *Y*_*k*_, also represents an average of the local Ca^2+^ sparks that may independently occur at the different spatial sites (see, for example, Nett et al., 2002). In such way, the variable *Y*_*k*_ represents a tonic effect of astrocytic activation on the mean field synaptic dynamics. We further refer *Y*_1_ as the concentration of astrocytic glutamate concentration modulating presynaptic release and *Y*_2_ as D-serine concentration modulating postsynaptic response of NMDA receptors.

In the mean field model, we estimated astrocytic modulation of the average concentration of neurotransmitter released. Thus, the average amount released for each incoming pulse was scaled with factor *k*_pre_= *k*_0_(1 + γ_1_*Y*_1_), where γ_1_ > 0 for the potentiation and γ_1_ < 0 for the depression, respectively. Thus, Equation (1) for presynaptic dynamics can be re-written as:

(8) $$\frac{dX}{dt}=-\alpha_{x}(X-k_{0}(1+\gamma_{1}Y_{1})H_{x}(I_{\text{pre}}-0.5)).$$

In addition to the presynaptic effect release of D-serine modulated EPSC through postsynaptic NMDARs. In the model, it was accounted for by the increase of the amplitudes of PSCs, *I*_EPSCs_, with:

(9) $$b=b_{0}\left(\text{1}+\gamma_{\text{2}}Y_{\text{2}}\right),$$

where γ_2_ is the gain of the D-serine effect.

**Figure 4:**
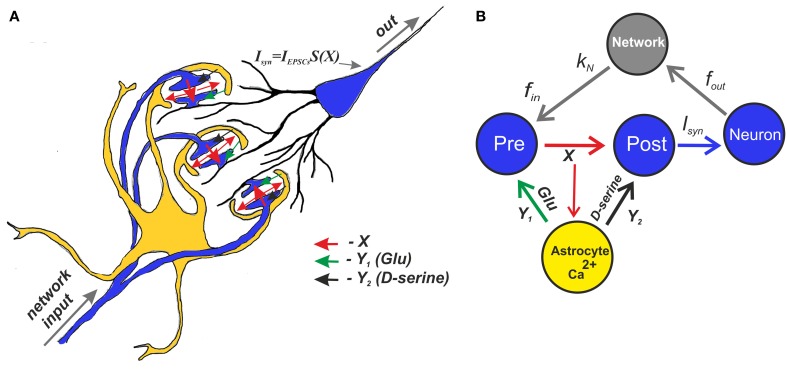
**A schematic view of the mean field model of synaptic transmission supplied with a network feedback. (A)** A network neuron has a large number of synaptic contacts. The presynapses were described by the mean field concentration of neurotransmitter (e.g., glutamate released by presynaptic terminals), *X*. The postsynapses were described by postsynaptic currents, *I*_EPSCs_. The local postsynaptic events were summarized and described by an integration function *S*(*X*), which reflects single synaptic events that have been filtered below the threshold. Astrocytic activation was accounted for by the transient increase of the mean field concentration of gliatransmitter (e.g., glutamate and D-serine). **(B)** A tripartite synapse with astrocytic feedback mediated by glutamate and D-serine provides activation of the postsynaptic neuron shown here as the mean field network neuron. The synapse is activated by an input spike train, *f*_in_. There is also spontaneous release rate, *f*_0_, that is independent of the input rate. In addition, the output spikes go through the network and return as separate inputs. The network impact is accounted for by the presence of the correlation, *k*_*N*_, between the output spiking rate and the input frequency.

Schematically, the mean field model of synaptic transmission is shown in Figure 4A.

### The firing rate coordination circuit

As a part of the neuronal network, the neuron is stimulated by signals generated by specific excitation transmission pathways and contributes to their sustainment by its own spikes. Because the synaptic architecture of even a simple neuronal network can be extremely complicated, it is very difficult to identify the precise spiking sequences generated by the network signaling circuits. Moreover, the same network may generate different sequences with variable interspike statistics. The repeatable spiking sequences found experimentally in both *in vivo* and *in vitro* conditions can serve as an example of such network behavior (Ikegaya et al., 2004). In the framework of the mean field approach, we followed the average frequency for the time scale up to seconds that is similar to a replay of the basic network signaling pathways. In such a consideration, a mean field neuron received a Poisson spike train input that fits the statistics of generally uncorrelated sources of input spikes, as we used in the synaptic transmission model. Next, we assumed that the mean field neuron contributes to the network activity by firing with a mean frequency, *f*_out_. The output signal from the neuron further propagates through the network in divergent/convergent signaling pathways and returns to the neuron in the form of separate inputs. In spontaneous network dynamics, the homeostatic states should be characterized by a reproducible activation. Perhaps the simplest model of such a network impact could be the presence of a correlation between the output and input firing states:

(10) $$f_{\text{in}}=f_{0}+k_{N}f_{\text{out}},$$

where *k*_*N*_ is the correlation coefficient determining the gain of the network control of a particular neuron, and *f*_0_ is the rate of the input-independent spontaneous presynaptic release.

For our input–output correlation model very simple predictions can be immediately derived from Equation (10) in limit cases. If *k*_*N*_ « 1, then the neuron is out of network feedback and its activity goes at low level induced by spontaneous release, *f*_0_. If *k*_*N*_ » 1, then the excitation circulation circuits are rapidly stimulating the neuron to its maximal hyperexcited state and may be considered as seizure-like dynamics. In the framework of our modeling approach, we described the network feedback variable *f* by the time scale parameter, τ_*N*_, and formulate the feedback using a first-order linear relaxation equation:

(11) $$\frac{df}{dt}=(k_{N}f_{\text{out}}+f_{0}-f)/\tau_{N}.$$

The solution of Equation (11) defines the input frequencies *f*_in_ = *f*(*t*) for simulation of the evoked responses.

Figure 4B shows a schematic illustration of the mean field model of synaptic transmission with network feedback.

The central element of the circuit is a network neuron that integrates EPSCs coming from a mean field synapse defined by Equations (1–11). The presynaptic dynamics is defined by the mean field concentration of the neurotransmitter released from the presynaptic terminals. This release is determined by the presynaptic spiking, *f*_in_, and spontaneous release, *f*_0_, incorporated in the presynaptic current *I*_pre_ to unify the model formalism. In the mean field approach, we assume that uncorrelated synaptic events occur at different spatial sites and modeled them by the Poisson distribution of event timings and by mean field variables in space. The astrocyte is represented by spatially distributed astrocytic processes that function independently to locally modulate synaptic dynamics. The Ca^2+^ transients in the astrocyte determined the concentration of gliotransmitters in the synaptic sites, which were determined by mean field concentration variables, *Y*_1_ (glutamate) and *Y*_2_ (D-serine). Overall, the neuronal response was characterized by the average spike frequency (Figure 2). The feedback is characterized by linear correlation between input and output firing rates according to Equation (11).

Constants and parameters used in simulations of Equations (1–11) are listed in Table 1.

**Table 1:** **Model parameters**.

**Parameter**	**Value**	**Description**
α_*x*_	0.1 ms^−1^	Neurotransmitter clearance constant
*k*_pre_	2	The efficacy of neurotransmitter release
α_*i*_	0.1 ms^−1^	Rate constant of EPSCs
*b*_0_	5–50	Scaling factor of gamma-distribution
θ_*x*_	0.2	Midpoint of activation function *S*(x) (Equation 5)
*k*_*x*_	0.05	Slope of activation function *S*(x) (Equation 5)
α_1_	0.01 ms^−1^	Clearance constant of glutamate released from astrocyte
α_2_	0.01 ms^−1^	Clearance constant of D-serine released from astrocyte
Θ_1,2_	0.3	Midpoint of gliatransmitter activation function *H*_1,2_(x) (Equation 7)
*k*_1,2_	0.1	Slope of activation function *H*_1,2_(x) (Equation 7)
γ_1_	−0.8	Presynaptic feedback gain describing the influence of astrocytic glutamate on the average amount of released neurotransmitter
γ_2_	0.4	Postsynaptic feedforward gain describing the influence of astrocytic D-serine on EPSCs amplitudes
*f*_0_	0.02–0.03 kHz	Frequency of spontaneous activation of the synaptic transmission
*k*_*N*_	3	Correlation coefficient determining the gain of the network feedback (Equation 10)
τ_*N*_	0.1 ms^−1^	Rate of network feedback

## Results

### Signal transmission in the tripartite synapse

**Figure 5:**
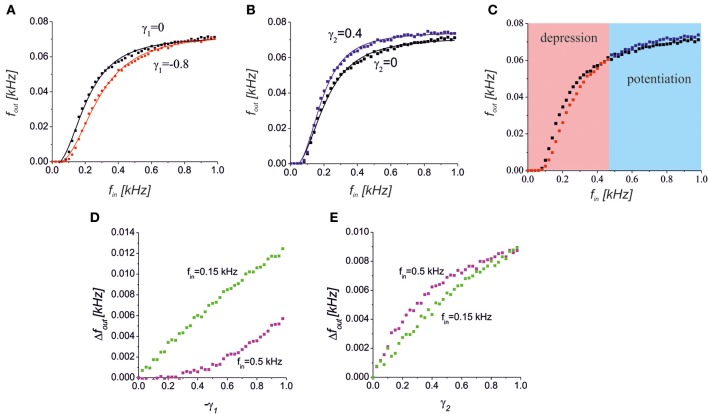
**(A)** A reduction of the presynaptic release caused a decrease in the spiking response in the middle frequency band. The black and red points with the corresponding logistic curve fits show a response without (γ_1_ = 0) and with (γ_1_ = −0.8) astrocytic activation feedback, respectively. **(B)** Increase of the response spiking rate due to the D-serine-mediated postsynaptic feedforward effect for γ_2_ = 0.4 (blue dots) relative to the control conditions (black dots). **(C)** The bidirectional effect of the astrocytic activation for both the presynaptic feedback and postsynaptic feedforward modulations for γ_1_ = −0.8, γ_2_ = 0.4. The red and blue areas show the frequency ranges when the response is depressed or potentiated, respectively. **(D,E)** The dependence of absolute output frequency changes Δ*f*_out_ = *f*_out_ − *f*_out_(γ_1,2_ = 0) on the gain of presynaptic depression, γ_1_, for γ_2_ = 0 **(D)** and on the gain of postsynaptic upscaling, γ_2_, for γ_1_ = 0 **(E)**. Green and magenta points correspond to input frequencies *f*_in_ = 0.5 kHz and 0.15 kHz, respectively.

We considered the dynamics of the signal transmission in the tripartite synapse for different input frequencies. We analysed a condition where the glutamate released from the astrocyte depresses neurotransmitter release (Semyanov and Kullmann, 2000). By setting γ_1_ < 0 in the model, we found that such a depression decreases the spiking response (Figure 5A). Importantly, the astrocytic feedback did not give any significant impact at the low and high input frequencies. For the low input frequencies, the probability of astrocytic activation was low (Pasti et al., 1997; Marchaland et al., 2008) and, thus, there was no gliotransmitter modulation of the presynaptic release. At the high input frequencies, the mean field concentration of the neurotransmitter reached its saturation level (all possible postsynaptic receptors were occupied) and the neuronal response was similar to that observed in the control condition without the astrocytic feedback.

Another effect of astrocytic activation was the D-serine-mediated potentiation of postsynaptic responses that we modeled by changes in the EPSCs amplitudes as a function of the gliotransmitter concentration *Y*_2_. Figure 5B illustrates the response curve in a model of the synapse, with γ_2_ > 0. The scaling of the EPSCs probability distribution caused a corresponding scaling of the spiking response curve. The mechanism of such scaling can be explained by D-serine mediated activation of postsynaptic NMDA receptors, and amplification of EPSCs amplitudes with the same level of occupancy of postsynaptic receptors with glutamate.

Interestingly, astrocytic activation can have both potentiating and depressing effects that contribute differently depending on the input frequency, if an astrocyte has both a reduction in neurotransmitter release and a postsynaptic upscaling of the EPSCs amplitudes (Figure 5C). Increasing the gain of presynaptic depression (−γ_1_) led to quite different absolute values of frequency change, Δ*f*_out_ = |*f*_out_ − *f*_out_(γ_1,2_ = 0)| for different intensities of the input (Figure 5D). For lower input frequency the impact of the γ_1_, i.e. Δ*f*_out_, was higher. The opposite situation was for the postsynaptic upscaling gain, γ_2_ (Figure 5E). The impact of γ_2_ is more significant for higher values of the input frequency. Note that for large values of γ_2_ the upscaling reached the saturation level (magenta dependence in Figure 5E).

### Network impact

**Figure 6:**
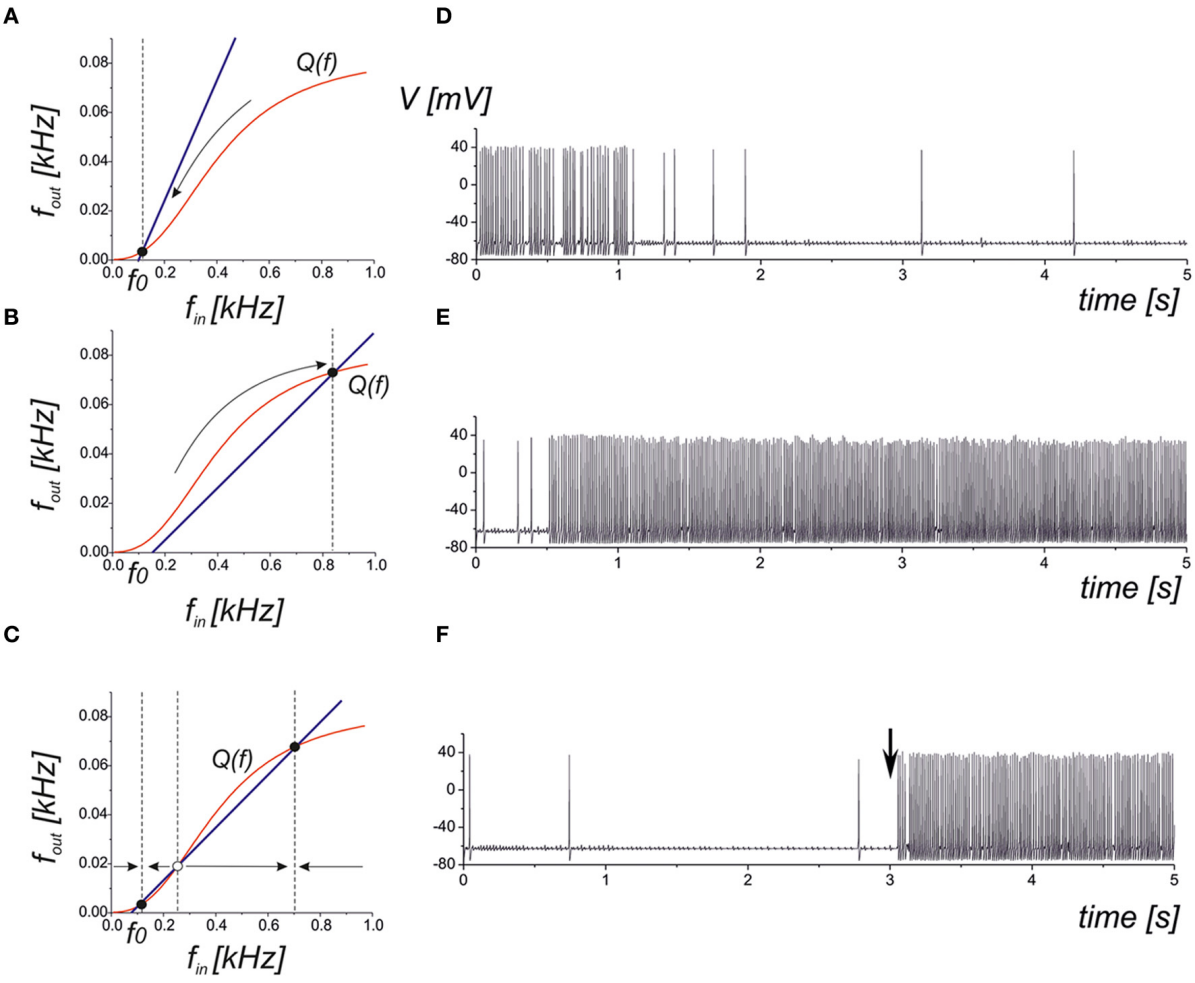
**Qualitative illustration of the network feedback dynamics in phase plane (*f*_in_, *f*_out_) and corresponding spiking sequences calculated from Equations (1–11)**. The curve *Q*(*f*) represents the input–output characteristics of the tripartite synapse (red curve). The blue line shows the network feedback correlating with the output frequency and the input spike train in Equation (10). The *f*_0_ is the frequency of spontaneous activation of the synaptic transmission. **(A)** Low activity mode. The neuronal dynamics are defined mainly by spontaneous firings. **(B)** High activity mode. The neuron fires at a high (close to saturation level) firing rate. **(C)** Bistable model. Low and high levels co-exist. Either level is realized depending on the initial conditions and/or due to the appropriate external stimulation. **(D)** Transition into spontaneous firing (panel **A**) for *k*_*N*_ = 0.1. **(E)** Transition into a higher activity state (panel B) for *k*_*N*_ = 5. **(F)** Bistability corresponding to phase plane in panel **(C)**. A stimulus in the form of a short high-frequency spike train injected into the input at *t* = 3 s induces the transition to a high activity level.

Because we are interested in the time averaged dynamics, it is important to estimate the steady-state functions of the network feedback. As we have illustrated in Figure 2, the neuronal response is converged to the input–output frequency curve, depending on the input frequency:

(12) $$f_{\text{out}}=Q\left(f_{\text{in}}\right),$$

where the function *Q*(*f*) can be approximated by a logistic curve (Figure 2). It is easy to determine that the network steady-state conditions will be given by the intersection points of the curves defined by Equations (10) and (12). There are three principle mutual arrangements of the steady-state curves (Figure 6). The level of network correlation is defined by the gain, *k*_*N*_, which determines the slope of the line (10), 1/*k*_*N*_, in the phase plane (*f*_in_, *f*_out_). If the network gain, *k*_*N*_, is small enough for low enough spontaneous activity, *f*_0_, then the curves have a single intersection point with low activity (Figure 6A). In the linear relaxation limit, e.g., assuming that the dynamics of relaxation to the curve (12) and to the line (10) are independent, we find that the steady-state will be locally stable and for any initial conditions, the neuron-to-network dynamics will converge upon the state of low activity mainly defined by the spontaneous synaptic activation component, *f*_0_. Figure 6D shows the evolution of the neuronal membrane potential in such conditions. The dynamics converges upon the state of low frequency and the network impact is negligible in this case. Figure 6B illustrates the opposite situation where there is a single intersection point in the upper branch of the frequency curve. Despite starting from low spontaneous activity, the network feedback brings the system to a relatively high spiking level (Figure 6E). The third alternative is a bistability when two stable states of low and high activity co-exist (Figure 6C). Depending on the initial conditions, the neuron may generate either high-frequency spiking, which is described as the “network-evoked” response or spontaneous firing. Interestingly, the application of a strong enough stimulus may come, for example, from another network group that may induce the switching of the neuron between the spontaneous and evoked modes, as illustrated in Figure 6F.

**Figure 7:**
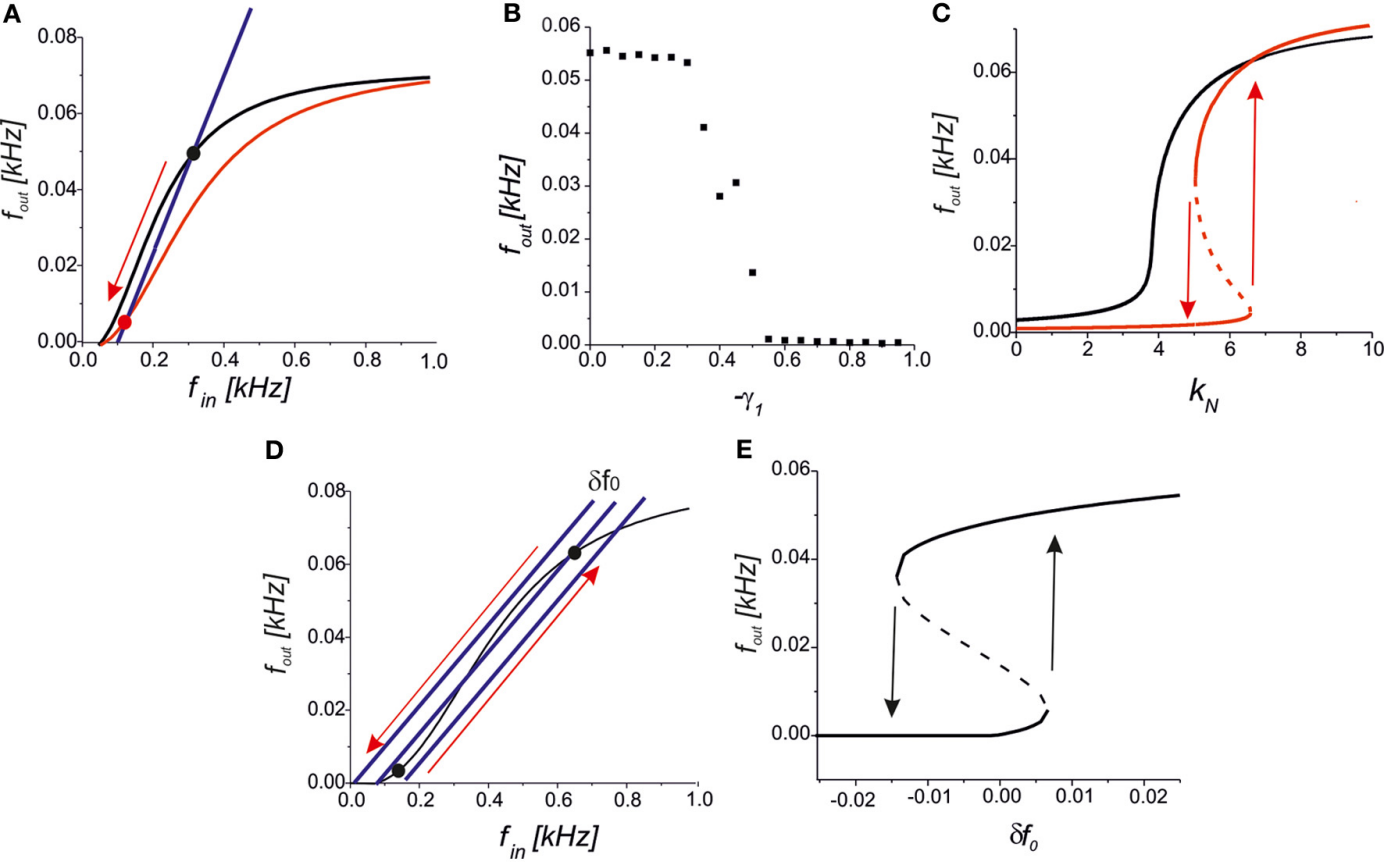
**(A)** A qualitative view on the presynaptic feedback in the tripartite synapse leading to the suppression of the spiking activity of the network neuron. **(B)** The dependence of the firing rate on the gain of presynaptic feedback (-γ_1_) for *f*_0_ = 0.09 kHz, *k*_*N*_ = 2.5, γ_2_ = 0. **(C)** Bifurcation diagram showing the output firing rate depending on the strength of the network feedback. For *f*_0_ = 0.07 kHz the dependence is monotonic in control conditions (black curve). Interval of bistability appears for the bi-directional astrocyte feedback with γ_1_ = −0.8, γ_2_ = 0.4 (red curves). **(D)** A qualitative view of the firing rate changes due to small fluctuations in the spontaneous EPSCs frequency, δ*f*_0_. **(E)** Bifurcation diagram of output firing states depending on δ*f*_0_ for *k*_*N*_ = 5, *f*_0_ = 0.05 kHz, γ_1,2_ = 0. Transitions between the low- and the high-activity states marked by arrows occur at the points of saddle-node bifurcations.

We further analysed the astrocytic impact on the synaptic transmission. In the steady-state approximation, we analysed the mutual arrangements of the curves under the influence of astrocytic feedback (Figure 5). A reduction of the presynaptic release may completely inhibit the activity by the network feedback in the middle frequency range (Figure 7A). A steady firing rate in control conditions (black dot in Figure 7A) shifts to a low firing level (red dot in Figure 7A) due to astrocytic activation. Increasing the gain of presynaptic depression above a critical value led to the transition to spontaneous firing defined by spontaneous release frequency *f*_0_ (Figure 7B). In other words, the neuron may be temporally excluded from a coordinated network firing, which may be protective mechanism from hyperexcitation. Next, activation of astrocytes may modulate network firing dynamics by the emergence of bistability of the high- and the low-frequency firing modes. Figure 7C illustrates the dependence of output firing rate on the strength of the correlation feedback. We assumed that without astrocytes the output rate was monotonic (black curve in Figure 7C). Bi-directional effect of astrocyte modulation leads to the appearance of two rate-encoded stable states of persistent neuronal firing for a certain range of feedback gains, *k*_*N*_ (red curves in Figure 7C). Importantly, the activation of astrocytes leads to two major modulation effects, as qualitatively demonstrated by the steady-state analysis. In particular, the threshold of the correlated firing leading to the high-activity state is changed due to the reduction of the neurotransmitter release probability. Thus, although models with a direct recurrent excitatory feedback can generate bistable neuronal firing (Koulakov et al., 2002; Goldman et al., 2003) in the absence of glial impact, the interval of bistability is broadening if the bi-directional effect of gliotransmitters on synaptic transmission is considered. In other words, in the presence of astrocytes the neuron can sustain its firing state (of high- or low-activity) for a wider range of network feedback, *k*_*N*_.

Several experimental studies have reported that astrocytic activation changes the frequency of spontaneous EPSCs (see, for example, Jourdain et al., 2007; Perea and Araque, 2007). An interesting prediction is derived from our model in terms of their influence on network dynamics. Assuming that the neuron output and input are correlated, as stated by Equation (10), we still have a network-independent parameter *f*_0_ describing the frequency of spontaneous presynaptic activation. For example, let the neuron state be tuned into its bistable mode as shown in Figure 7D and set to its spontaneous firing mode with low activity. Then, even a small transient increase in spontaneous frequency, *f*_0_ + δ *f*_0_, may occur due to astrocytic activation, which leads to a transition to the high-activity state. Generally, the backward transition with decreasing spontaneous frequency, *f*_0_ − δ *f*_0_, inhibits the neuron, excluding it from coordinated network firing (Figure 7D). Figure 7E illustrates the transitions between the low- and the high-activity states on a bifurcation diagram depending on the fluctuations of spontaneous frequency, δ *f*_0_.

**Figure 8:**
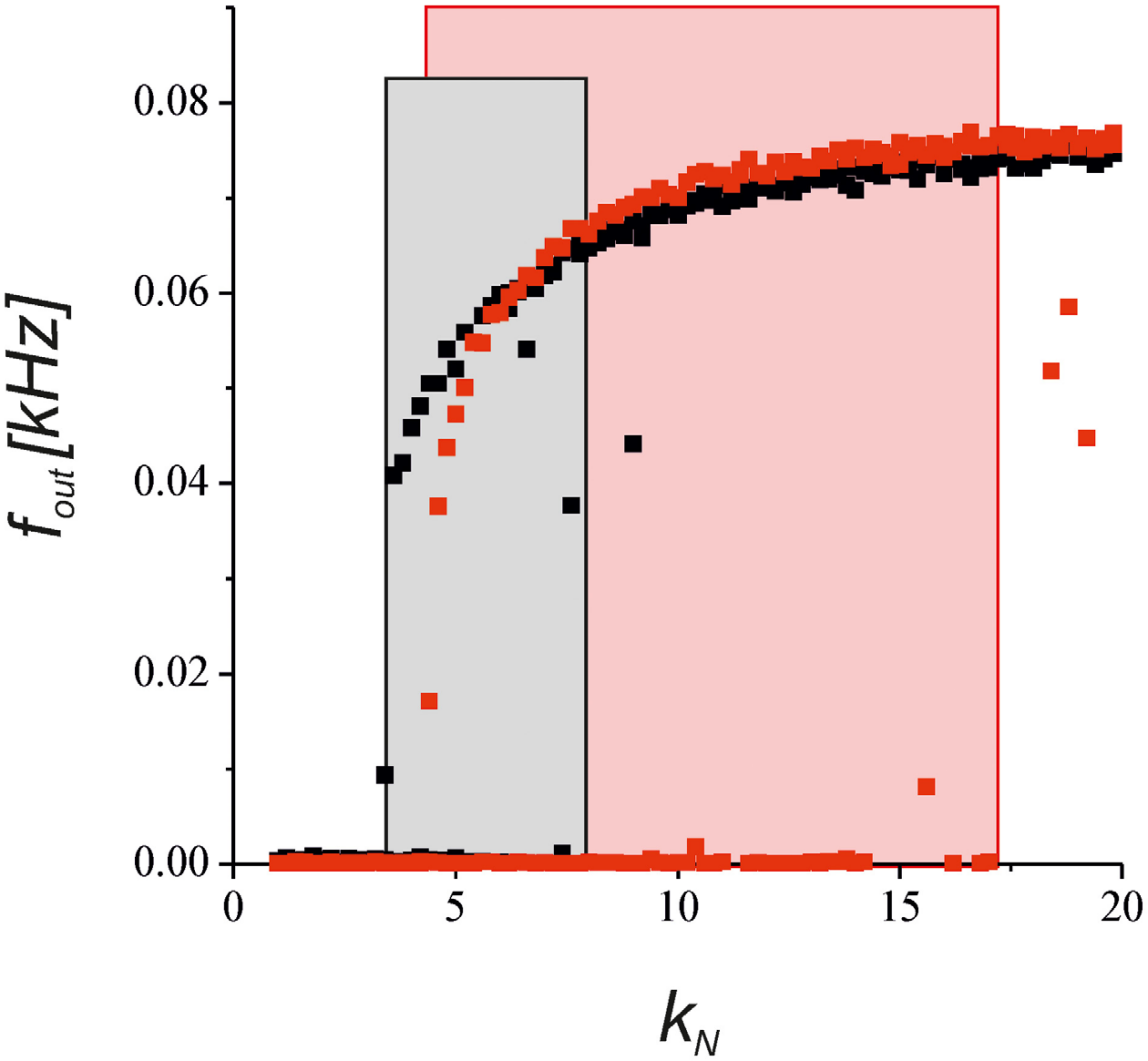
**The dependence of the neuronal firing rate on the network feedback gain, *k*_*N*_, in Equations (1–11)**. The black points show the results for the synapse without the astrocytic feedback. The red dots illustrates the modulation by the astrocyte in the tripartite synaptic transmission for γ_1_ = −0.8 and γ_2_ = 0.4. The rectangular areas show the intervals of bistable dynamics where the two stable steady states for low and high activities co-exist. The model was simulated for the different initial conditions [low and high values of *f*_in_ (*t* = 0)] for each value of *k*_*N*_.

To verify the predictions of the steady-state approximation, we simulated the model with a complete equation set to show how the output activity depends on the network impact for the synaptic transmission. Figure 8 shows a bifurcation diagram of the average spiking rate depending on the network feedback gain, *k*_*N*_. Increasing the gain led to a bistable dynamics marked by rectangle areas. Astrocyte activation shifted the boundary of bistability due to the depression of presynaptic release and enlarged the bistability interval due to the bi-directional regulation effects (red points in Figure 8).

## Discussion

We developed a computational model of astrocytic regulation of synaptic transmission predicting that the astrocytes can effectively modulate neuronal network firing. We considered a mean field network neuron. Network impact was modeled by a certain level of correlation between its output and input. Thus, the presynaptic dynamics consisted of spontaneous activity and the activity induced by the network feedback. The postsynaptic dynamics was modeled by a number of PSCs integrating at soma and leading to a spike generation. The astrocytic compartment was characterized by local release of glutamate and D-serine modulating the presynaptic release probability and the postsynaptic amplitudes of EPSCs, respectively.

Here we analysed only two potential effects of astrocyte feedback on the synapse: presynaptic depression of glutamate release and postsynaptic enhancement of these responses. These phenomena correspond to reports showing that astrocyte-released glutamate can reduce the release probability of neurotransmitter (Araque et al., 1998) while astrocytic release of D-serine enhances the response of postsynaptic NMDA receptors (Henneberger et al., 2010). Because the number of synapses to the target cell is finite, this limits the maximal amount of synaptic inputs, which can be simultaneously activated. Thus, the cell input still can be saturated even with decreased release probability by the increase in *f*_in_. However, when *f*_in_ is low and does not saturate cell input, reduced release probability decreases *f*_out_. The postsynaptic effect of gliotransmitter D-serine is principally different. D-serine is co-agonist of NMDA receptors and still required even if these receptors are bound to glutamate. Thus, D-serine increases the response of the postsynaptic cell to the same amount of glutamate (even saturating) because of additional recruitment of postsynaptic NMDA receptors. It allows a larger *f*_out_ at saturating conditions at large *f*_in_. When these presynaptic and postsynaptic effects coincide, *f*_out_ is reduced at low *f*_in_ because of a reduced release probability, but increases at a high *f*_in_ because of the increase in the level of saturation. Thus, simultaneous recruitment of two counteracting types of astrocytic modulation actually works as a high-pass filter. A similar phenomenon has been reported in hippocampal slices for glutamate acting presynaptically on both mGluRs and kainate receptors (Kullmann and Semyanov, 2002). mGluRs reduce the release probability, while kainate receptors increase presynaptic excitability. Co-activation of both types of receptors contrasted action potentials dependent synaptic GABA release versus spontaneous action potential independent release in CA1 interneurons. The dependence of the astrocytic effects on the density of receptors targeted by a gliotransmitter and on the efficiency of gliotransmitter clearance opens up the possibility that a relative weight of each influence is not a constant. These weights can be represented as a vector of parameters of gliotransmitter influence specific to the particular synapse. These vectors can be different for different synapses and determine differential modulation at different synapses by an equivalent amount of released gliotransmitter. Similar bi-directional changes in the efficacy of signal transmission by astrocytic modulation were recently found by De Pittà et al. (2011). They showed that gliotransmitter acting on presynaptic site can regulate the release differentially depending on frequency of input signal.

An interesting consequence of the proposed model for network computations is that such differential modulation can be effective in controlling network firing states. Indeed, we found that even small changes in the input–output function induced by transient astrocyte activations when superimposed with network feedback may lead to dramatic changes in neuron firing. In particular, the astrocyte may activate or deactivate specific neurons to be involved in network firing. Another interesting effect is the enforcement of the bistability by the astrocyte. Coexistence of two stable firing modes at the level of single neuron implies coexistence of multiple rate-encoded states in spiking networks. In such a treatment, the astrocytes may serve not only as local gate-keepers in synaptic transmission (Volman et al., 2006) but also as activity guiders coordinating information processing at the network level (Semyanov, 2008).

### Conflict of interest statement

The authors declare that the research was conducted in the absence of any commercial or financial relationships that could be construed as a potential conflict of interest.
